# Magnetic field evolution in $\mathrm{Au+Au}$ collisions at $\sqrt{s_{NN}} = {200}\;{\text{GeV}}$

**DOI:** 10.1038/s41598-023-48705-1

**Published:** 2023-12-06

**Authors:** Chun-Hui Zhang, Qi-Chun Feng, Yan-Yu Ren, Li-Ming Hua, Lei Huo

**Affiliations:** School of Physics, Harbin Institute of Technology, Harbin, 150001 China

**Keywords:** Mathematics and computing, Physics

## Abstract

In high energy heavy-ion collisions, the high speed valence charges will produce intense electromagnetic fields within the resulting quark-gluon plasma. Utilizing the AMPT model, this paper presents a comprehensive analysis of the magnetic field distribution generated from non-central collisions between $\mathrm{Au+Au}$ nuclei at $\sqrt{s_{\text{NN}}}={200}\;{\text{GeV}}$. The initial geometric parameters of the collision and the electric conductivity of the quark-gluon plasma have a dominant influence on the evolution of the magnetic field, while the plasma diffusion and the CME effect have a lesser impact and only slightly involve the original magnetic field by inducing new magnetic fields. This finding suggests that the dynamics of the quark-gluon plasma can be roughly decoupled from the effect of the electromagnetic field.

## Introduction

Relativistic heavy-ion collisions can produce high-speed charged particles that generate immense electromagnetic fields^1,2^. Recent experimental measurements and numerical simulations suggest that collisions between $\mathrm{Au+Au}$ nucleis at the energy of $\sqrt{s_{\text{NN}}}={200}\;{\text{GeV}}$ in the BNL Relativistic Heavy Ion Collider (RHIC) can produce magnetic field intensities of $10^{18}{-}10^{19}$ Gauss^3^. Furthermore, $\mathrm{Pb+Pb}$ collisions at $\sqrt{s_{\text{NN}}}={2.76}\; {\text{TeV}}$ in the CERN Large Hadron Collider (LHC) can produce magnetic field intensities of up to $10^{20}$ Gauss^4,5^. These fields are significantly stronger than the squared mass of electrons and light quarks $m_e^2, m_u^2, m_d^2$ and can strongly influence the dynamic processes of quark-gluon plasma substances^6,7^. In particular, one important aspect of these magnetic fields is their ability to cause anomalous transport phenomena in heavy ion collisions, such as the chiral magnetic effect (CME)^8–10^, the chrial separation effect (CSE)^11,12^, and the chiral magnetic wave (CWM)^13,14^. The non-trivial topological fluctuations of gluons in the QCD vacuum can break the parity and charge conjugation parity symmetry in the strong interaction. When a strong external magnetic field is present, fermions with different chirality separate along the direction of the magnetic field to produce a vector current, which is called CME. Experimental studies on anomalous transport have been carried out in RHIC and LHC, and the measured results provide signals consistent with CME predictions^15–18^. The study of these phenomena has important implications for understanding of the properties of QGP under extreme conditions and the research for new physics.

To accurately understand how the magnetic field interacts with the quark-gluon plasma produced in high-energy collisions, it is essential to study the evolution of the magnetic field existing in the thermally expanded nuclear medium. The spatial distributions of the electromagnetic fields and the electromagnetic anomaly $\varvec{E}\cdot \varvec{B}$ in $\mathrm{Au+Au}$ collisions have been reasonably calculated at the RHIC energy based on a multi-phase transport (AMPT) model^19^. Additionally, some investigations explore the space-time evolution of electromagnetic fields and azimuthal angle fluctuations of these fields and their correlation with the initial matter geometry^20,21^. Previous studies also suggest that the magnetic field originates from the spectators of the collision nucleus, appears only at the preliminary time of collision, and then decays and disappears quickly within ${0.5} \; {\text {fm}}/c$ as the spectators fly away from the reaction region^22–25^. But the QGP generated in the collision contains abundant conductive sea quarks that can respond to the magnetic field. Since the QGP behaves like a nearly ideal fluid, its response to the magnetic field can be measured through transport coefficients such as the electric conductivity and the chiral conductivity. Therefore, the calculations involving the magnetic field must take these coefficients into account. Research on the magnetofluid equation of ideal fluids under strong electromagnetic fields has shown that the electromagnetic field can affect both the ideal flow and the transport coefficients of plasma^20,26–28^, therefore, it is necessary to verify that the kinetic coefficients do not heavily depend on the magnetic field.

At the early stages of heavy-ion collisions, gluons primarily make up the produced partonic matter, which exists in a state far from equilibrium. The QGP can be approximated as an insulator, during this particular period, which includes the time before the collision, the magnetic field in space can be determined by Lienard-Wiechert potentials^22^. As time progresses, more quarks and anti-quarks are excited and produced, leading to the QGP approaching thermal equilibrium. Despite the lack of a quantitative theory to fully comprehend the thermalization problem, phenomenological studies have demonstrated that the completion time for thermalization is relatively brief compared to the overall lifetime of QGP, and the evolution of the thermalized QGP can be well described by ideal fluid mechanics at this stage. According to the lattice QCD theory, the QGP is a good conductor, with an important transport coefficient known as the electic conductivity $\sigma$. The perturbative QCD calculation shows that at very high temperatures, the electic conductivity is $\sigma \approx 6T/e^2$^29^. Recently, lattice QCD calculations with $N_{f}=2+1$ dynamical Wilson fermions obtained that $\sigma \approx 0.1C_{EM}T{-}0.3C_{EM}T$^30,31^ for temperature $T_{c}<T<2T_{c}$, where *T* and $C_{EM}$ are the deconfinement temperature and the EM vertex parameter. At $T={350} \; \; {\text {MeV}}$, the resulting $\sigma$ is about ${5}\; {\text {MeV}}$. Furthermore, we only focus on the chiral magnetic effect, which can be described as $\varvec{j}=\sigma _\chi \varvec{B}$, and

1 $$\begin{aligned} \sigma _\chi = \mu _{5}\frac{e^2}{2\pi ^{2}}N_{c}\sum _{f}q_{f}^{2} \end{aligned}$$

is the chiral conductivity induced by the QED anomaly^9^.

In our work, the diffusion process of the quark-gluon plasma is treated as a perturbation to estimate its contribution to the magnetic field. This approach allows for a better understanding of the complex interactions between the magnetic field and the QGP medium produced in high-energy collisions. We employ the AMPT model to simulate the collision with impact parameter $b={7} \; {\text {fm}}$ between $\mathrm{Au+Au}$ nuclei at $\sqrt{s_{\text{NN}}}={200} \; {\text{Gev}}$. We calculate the space-time distribution of magnetic fields, taking into account the electric conductivity, the diffusion of plasma, and chiral magnetic effect. On this basis, the impact of plasma diffusion and CME on the magnetic field is investigated. In our calculations, we assume that the nuclear matter in the collision region is a homogeneously diffused plasma with a velocity distribution that is obtained by solving the ideal hydrodynamic equation. We also assume that the plasma has a constant electric and chiral conductivity, which enables us to obtain analytical results.

## Methods

The AMPT model^32–36^ can well characterizes complicated dynamic transport processes in heavy-ion collisions, which effectively supports our the magnetic field distribution calculations. To accurately describe the cases when the QGP is produced, we choose the string melting version^34,37^ which encompasses important evolution stages of collisions, including initial state, parton cascade, hadronization, and hadron rescatterings. At the initial stage, two $\text{Au}$ nuclei collide head-on at a certain speed $\varvec{v}$ along the *z* axis in the center of mass system. After the collision, the AMPT model tracks the trajectory of each nucleon to determine whether there was a collision and what type of collision occurred. Nucleons experiencing the inelastic collision subsequently participate in the parton cascade and contribute to the formation of quark-gluon plasma, as well as those that do not experience any collision will fly away from the center region of collision, constantly radiating magnetic field. The initial distribution of nucleons in the nucleus before the collision is given by the Woods-Saxon distribution:

2 $$\begin{aligned} \rho (r)=\frac{\rho _0}{1+\exp ((r-R_0)/a)} \end{aligned}$$

where $\rho _0={0.17} \; {\text {fm}^{-3}}$, $a={0.535} \; {\text {fm}}$, and $R_{0}={6.5}\; {\text {fm}}$ is the radius of the incoming $\text{Au}$ nucleus.

The Maxwell equations that describe the evolution of electromagnetic fields in a conductive plasma in diffusive thermal equilibrium can be written as^22^:

3a $$\begin{aligned}&\nabla \cdot \varvec{E}=\rho \end{aligned}$$

3b $$\begin{aligned}&\nabla \times \varvec{E}=-\partial _t \varvec{B} \end{aligned}$$

3c $$\begin{aligned}&\nabla \cdot \varvec{B}=0 \end{aligned}$$

3d $$\begin{aligned}&\nabla \times \varvec{B}=\partial _t \varvec{E}+\sigma (\varvec{E}+\varvec{u}\times \varvec{B})+\sigma _\chi \varvec{B}+\varvec{J}_{ext} \end{aligned}$$

Where $\varvec{u}$ is the velocity distribution of plasma diffusion. $\sigma (\varvec{E}+\varvec{u}\times \varvec{B})$ and $\sigma _\chi \varvec{B}$ represent internal currents induced by Ohm’s law^22^ and CME, respectively. $\varvec{J}_{ext}=\sum _{N}\varvec{j}$ denotes the external current generated by the motion of total nucleus, where *N* is the number of unwounded protons, and consider the current produced by a point charge *e* moving along the *z*-axis with velocity *v* in cylindrical coordinate $(\hat{b},\hat{\phi },\hat{z})$ as:

4 $$\begin{aligned} \varvec{j}=ev\hat{\varvec{z}}\delta (\varvec{b})\delta (z-vt) \end{aligned}$$

By utilizing Eq. (2), we can rewrite the Maxwell equations into equivalent magnetohydrodynamic equations:

5 $$\begin{aligned}&(\partial ^2+\sigma \partial _t)\varvec{B}(t,\varvec{r})-\sigma _\chi \nabla \times \varvec{B}(t,\varvec{r}) -\sigma \nabla \times (\varvec{u}\times \varvec{B}(t,\varvec{r}))=\nabla \times \varvec{J}_{ext}(t,\varvec{r}) \end{aligned}$$

The equation contains the convection term of the magnetic field itself and the diffusion term of the coupling between the magnetic field and the flow of QGP, making it challenging to obtain direct analytical solutions in mathematics. In experiments, the detected particles are primarily located in the central rapidity range $|\eta |\leqslant 1$^38^, which imposes a condition for the diffusion of these particles: the velocity of the diffusive plasma $|\varvec{u}|\ll 1$. Furthermore, the chiral magnetic effect is weak, and it remains challenging to separate the CME signal from the complex electromagnetic background in experiments. In other words, the effect of the plasma flow and the CME on the magnetic field is only a perturbation compared to $\varvec{J}_{ext}$, and employing $\varvec{B}=\varvec{B}^{(0)}+\varvec{B}^{(1)}+\varvec{B}^{(2)}$, the Eq. (5) can be rewritten as follows:

6a $$\begin{aligned}&(\partial ^2+\sigma \partial _t)\varvec{B}^{(0)}(t,\varvec{r})=\nabla \times \varvec{J}_{ext}(t,\varvec{r}) \end{aligned}$$

6b $$\begin{aligned}&(\partial _t^2+\sigma \partial _t)\varvec{B}^{(1)}(t,\varvec{r})=\sigma \nabla \times (\varvec{u}\times \varvec{B}^{(0)}(t,\varvec{r})) \end{aligned}$$

6c $$\begin{aligned}&(\partial _t^2+\sigma \partial _t)\varvec{B}^{(2)}(t,\varvec{r})=\sigma _\chi \nabla \times \varvec{B}^{(0)}(t,\varvec{r}) \end{aligned}$$

The first of these equations describes the field created by the external currents in the stationary plasma, while the other two equations consider the plasma flow and the CME, respectively. We obtain three equations with a uniform form, and they share the same retarded Green’s function:

7 $$\begin{aligned} (\partial ^2+\sigma \partial _t)G(t,\varvec{r})=-\delta (\varvec{r}-\varvec{r}^{\prime })\delta (t-t^{\prime }) \end{aligned}$$

The retarded Green’s function is associated with the thermal diffusion equation^39^, which includes a dissipation term in the medium. An analytical solution for this equation can be obtained as follows:

8a $$\begin{aligned} G(\tau ,R)&=G_a(\tau ,R)+G_b(\tau ,R) \end{aligned}$$

8b $$\begin{aligned} G_a(\tau ,R)&=\frac{1}{4\pi }e^{-\frac{1}{2}\sigma \tau }\frac{\delta (\tau -R)}{R} \end{aligned}$$

8c $$\begin{aligned} G_b(\tau ,R)&=\frac{1}{4\pi }e^{-\frac{1}{2}\sigma \tau } \frac{\sigma /2}{\sqrt{\tau ^2-R^2}}I_1(\frac{\sigma }{2}\sqrt{\tau ^2-R^2})\theta (\tau -R) \end{aligned}$$

the variables $\tau =t-t^{\prime }$ and $R=|\varvec{r}-\varvec{r}^{\prime } |$ represent the time and position differences between field point and source point. Through the retarded Green’s function, the particular solutions of the corresponding magnetic fields are calculated as:

9a $$\begin{aligned} \varvec{B}^{(0)}(t,\varvec{r})=&\sigma \int \text{d}t^{\prime }\text{d}V^{\prime } G(t,\varvec{r}|t^{\prime },\varvec{r}^{\prime })\varvec{J}_{ext}(t^{\prime },\varvec{r}^{\prime }) \end{aligned}$$

9b $$\begin{aligned} \varvec{B}^{(1)}(t,\varvec{r})=&\sigma \int \text{d}t^{\prime }\text{d}V^{\prime }G(t,\varvec{r}|t^{\prime },\varvec{r}^{\prime })\nabla \times (\varvec{u}(t^{\prime },\varvec{r}^{\prime }) \times \varvec{B}^{(0)}(t^{\prime },\varvec{r}^{\prime }-\varvec{r}^{\prime \prime })) \end{aligned}$$

9c $$\begin{aligned} \varvec{B}^{(2)}(t,\varvec{r})=&\sigma _\chi \int \text{d}t^{\prime }\text{d}V^{\prime } G(t,\varvec{r}|t^{\prime },\varvec{r}^{\prime })\nabla \times \varvec{B}^{(0)}(t^{\prime },\varvec{r}^{\prime }-\varvec{r}^{\prime \prime }) \end{aligned}$$

Like the retarded Green’s function is expressed as a sum of two terms, we set $\varvec{B}^{(0)}=\varvec{B}_{a}^{(0)}+\varvec{B}_{b}^{(0)}$ and get

10 $$\begin{aligned} \varvec{B}_{a}^{(0)}(t,\varvec{r})=&\sum _{N}\frac{ev}{4\pi }\hat{\varvec{\phi }} \left (\frac{\sigma b}{2\xi ^2}+\frac{b}{\gamma ^2\xi ^3} \right) \times \exp \left[ -\frac{\sigma \gamma ^2}{2}(\xi -v\sqrt{\xi ^2-b^2/\gamma ^2})\right] \end{aligned}$$

where we set $\xi =\sqrt{(vt-z)^2+b^2/\gamma ^2}$.

For $G_b(\tau ,R)$, we note that $\sqrt{\tau ^2-R^2}\leqslant 2b/\gamma \ll 1$ and the virtual Bessel function $I_{1}(x)\approx x$ when $x\ll 1$. Thus, the magnetic field $\varvec{B}_{b}^{(0)}$ is expressed approximately as

11 $$\begin{aligned} \varvec{B}_{b}^{(0)}(t,\varvec{r}) \approx&\sum _{N}\frac{evb\sigma ^2}{16\pi }\hat{\varvec{\phi }}\frac{1}{\xi } \times \exp \left[ -\frac{\sigma \gamma ^2}{2}(\xi -v\sqrt{\xi ^2-b^2/\gamma ^2})\right] \end{aligned}$$

The field $\varvec{B}^{(0)}_{a}$ represents the retarded pulse field, which emerges due to the equivalent current in the plasma as a result of valence charges, and $\varvec{B}^{(0)}_{b}$ denotes the magnetic field generated by the excitation current in the QGP region after the collision. Although the contribution of $\varvec{B}^{(0)}_{b}$ to the total magnetic field is relatively weak since it is more than $\varvec{B}^{(0)}_{a}$ scaled by a factor of $\sigma$. However, in the late stage of the collision $\xi \gg 4/\sigma$ and $\xi \sim t$, it will take on a crucial role as it falls off as 1/*t*, whereas $\varvec{B}^{(0)}_{a}$ as $1/t^2$.

Furthermore, it is important to recognize that before the emergence of the quark-gluon plasma, a magnetic field was already present in space due to the movement of the colliding nuclei through the vacuum. At the moment of collision $t={0}\;\mathrm{fm/c}$, the magnetic field within the nuclear medium must correspond to the magnetic field that has existed in the interaction region prior to the emergence of the QGP. This work has been already done in Ref. ^40^. Subsequently, the initial magnetic field that persists within the medium after the collision is:

12 $$\begin{aligned} \varvec{B}_{init}(t,\varvec{r}) =&\sum _{N}\frac{ev\gamma }{4\pi }\hat{\varvec{\phi }}\int _{0}^{\infty } \text{d}k_{\bot } k_{\bot }J_{1}(k_{\bot }b) \times \exp (-k_{\bot }^{2}t/\sigma -k_{\bot }\gamma |z-vt_{0} |) \end{aligned}$$

Actually, it is the Dirichlet-type and Neumann-type boundary conditions associated with the Green’s function. Since $\varvec{B}_{init}$ is independent of the plasma diffusion and CME, we regard $\varvec{B}_{init}$ as a component of $\varvec{B}^{(0)}$, Thus, the complete solution to Eq. (6a) takes form $\varvec{B}^{(0)}=\varvec{B}_{a}^{(0)}+\varvec{B}_{b}^{(0)}+\varvec{B}_{init}$.

By using Eqs. (10) and (11), we can calculate the magnetic fields $\varvec{B}^{(1)}$ and $\varvec{B}^{(2)}$ by employing a numerical solution. However, prior to this, it is necessary to introduce a hydrodynamic model to depict the diffusion of quark-gluon plasma. To accomplish this, we adopt the method described in Ref.^41^, which involves assuming that the initial entropy density profile in the transverse plane follows a Gaussian distribution:

13 $$\begin{aligned} s(x,y)=s_{0}\exp \left (-\frac{x^{2}}{2a_{x}^{2}}-\frac{y^{2}}{2a_{y}^{2}} \right) \end{aligned}$$

where $a_{x}$ and $a_{y}$ denote the root-mean-square widths of the transverse plane of the QGP, and their magnitudes are in agreement with the radius of the collision nucleus. In the case of an $\mathrm{Au+Au}$ collision, the available calculation indicates that for $b={0} \; {\text {fm}}$, $a_{x}\sim a_{y}\sim {3} \; {\text {fm}}$, while for $b={10}\; {\text {fm}}$, $a_{x}\sim {2} \; {\text {fm}}$, $a_{y}\sim {3}\; {\text {fm}}$. By considering Bjorken expansion in the longitudinal direction $u_{z}=\frac{z}{t}$, it is possible to solve the ideal hydrodynamic equation of transverse diffusion and obtain the transverse velocity distribution of the plasma:

14 $$\begin{aligned} u_{x}=\frac{c_{s}^{2}}{a_{x}^{2}}xt \end{aligned}$$

15 $$\begin{aligned} u_{y}=\frac{c_{s}^{2}}{a_{y}^{2}}yt \end{aligned}$$

where $c_{s}=\sqrt{\partial P/\partial \varepsilon }$ is the speed of sound. Substituting the velocity fields into Eq. (9b), we can solve out $\varvec{B}^{(1)}$ numerically.

## Discussion

For the convenience of analysis and comparison with other studies, we convert our results to the cartesian coordinate system through the coordinate transformation $(\hat{b}, \hat{\phi }, \hat{z})\rightarrow (\hat{x}, \hat{y}, \hat{z})$, and the *z* axis corresponds to the direction of nuclear motion, and the *x* axis corresponds to the collision parameter direction. We simulate $\mathrm{Au+Au}$ collision at $\sqrt{s_{NN}}={200}\; {\text{GeV}}$ with impact parameter $b={7} \; {\text {fm}}$, and set electric conductivity $\sigma ={5.0}\; {\text {MeV}}$. The numerial results of our magnetic field calculations are discussed in the following.

**Figure 1:**
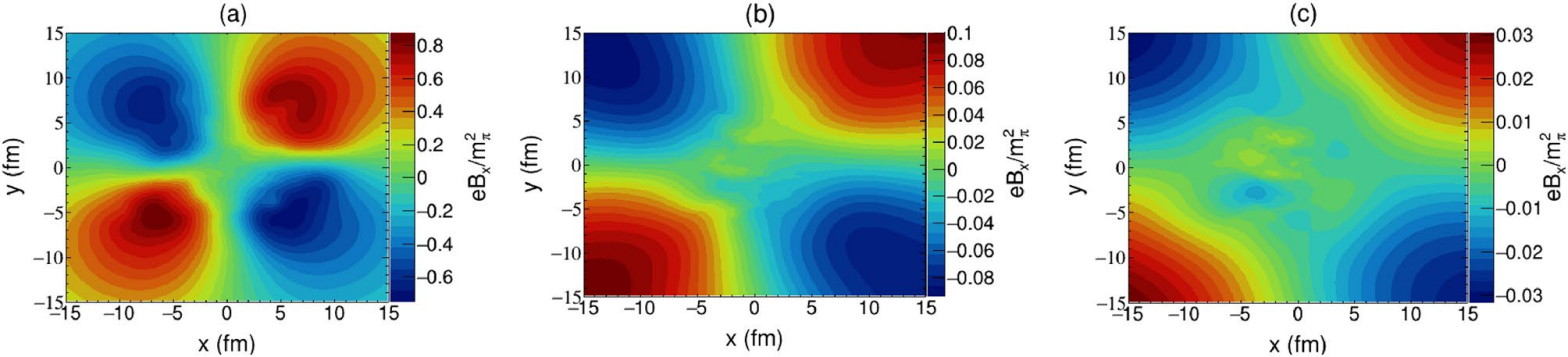
The spatial distribution of magnetic field $B_x$ on $x{-}y$ plane with $z=0$, calculated at $b = {7}\; {\text {fm}}$ and $\sqrt{s_{NN}} = {200}\; \; {\text{GeV}}$, (**a**) $t={0.3}\; {\text {fm}}/c$, (**b**) $t={1.0}\;{\text {fm}}/c$, and (**c**) $t={3.2}\;{\text {fm}}/c$.

**Figure 2:**
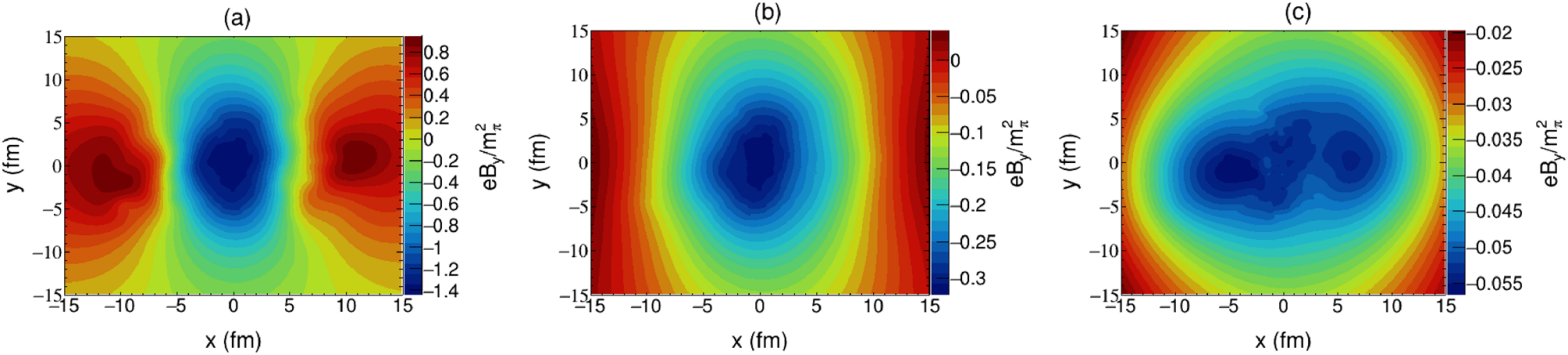
The spatial distribution of magnetic field $B_y$ on $x{-}y$ plane with $z=0$, calculated at $b = {7}\; {\text {fm}}$ and $\sqrt{s_{NN}} = {200}\; \; {\text{GeV}}$, (**a**) $t={0.3}\; {\text {fm}}/c$, (**b**) $t={1.0} \; {\text {fm}}/c$, and (**c**) $t={3.2} \; {\text {fm}}/c$.

Due to the geometric symmetry of the collision, the magnetic field in the center of the overlapp region is perpendicular to the reaction plane. However, in an actual collision event, the distribution of nucleons in the two colliding nuclei may not be the same, which could destroy this geometric symmetry and cause fluctuations of the magnetic field in the *x* direction. Figures 1 and  2 respectively illustrate the spatial distribution of magnetic fields $B_x$ and $B_y$. The overall spatial distribution of the magnetic field is fairly inhomogeneous at the very beginning of the nuclear collision. The magnetic field $B_x$ begins with a rotational symmetric structure, quickly spreads into surrounding space and reduces to zero. Compared to $B_y$, the stength of $B_x$ is much smaller in the geometric central region of collision where the nuclear matter is located, which should not significantly affect the QGP’s evolution. Relatively, the maximum value of magnetic field $B_y$ is emanated at the center region, which also expands and decreases in the transverse plane over time. After the collision has occurred for ${3}\; {\text {fm}}/c$, it gradually evolves into two extreme values that are related to the spectator nucleons. Our findings are generally in agreement with those obtained in Ref. ^2^. However, the time scale of the transition of magnetic field $B_y$ calculated by us, from one maximum to two extremes, is longer, which is the result of taking into account the quark-gluon plasma medium effect. On this condition, a sufficient response time is provided for the emergence of quantum anomalous transport phenomena. It can also be observed that the magnetic field in the transverse region of $r<{4} \; {\text {fm}}$ demonstrates a high homogeneity and constancy at the later stage of collision, which enables us to make some simplifying assumptions when estimating the phenomenological interaction between magnetic field and QGP.

**Figure 3:**
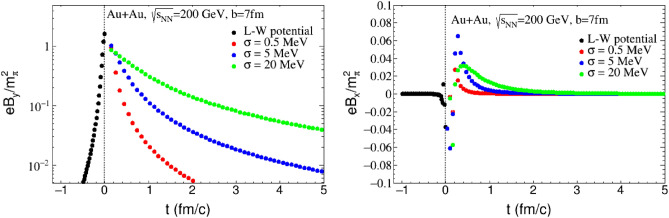
The time evolution of magnetic field at the point $\varvec{r}=0$ for $\mathrm{Au+Au}$ nucleis at $\sqrt{s_{NN}}={200}\; {\text{GeV}}$ and $b = {7} \; {\text {fm}}$. The left panel is for $B_y$ and the right panel is for $B_x$.

**Figure 4:**
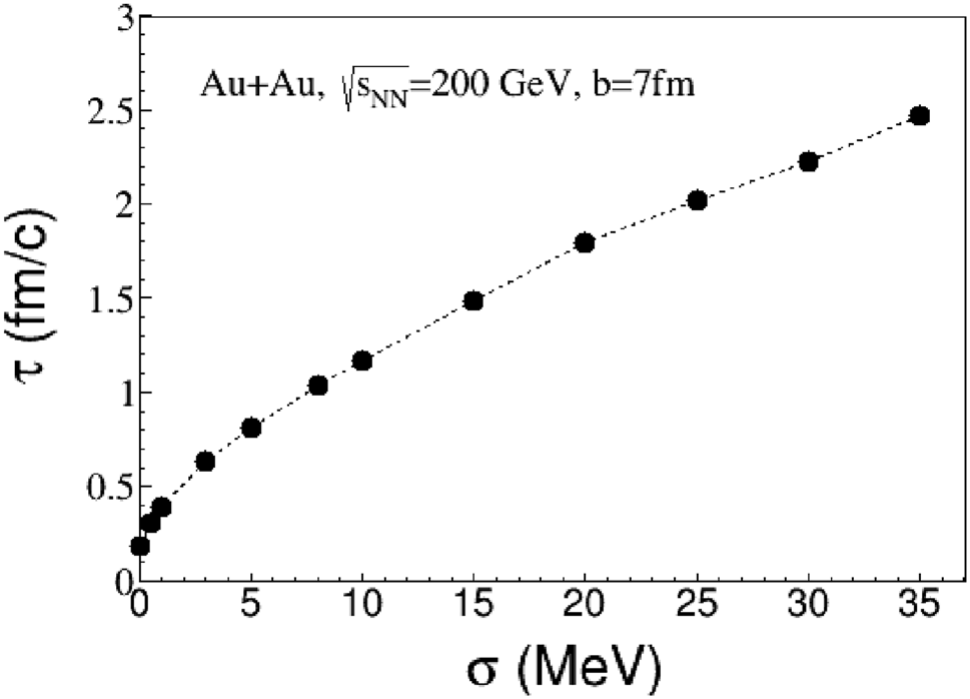
The lifetime of magnetic filed $B_y$ at $b = {7} \; {\text {fm}}$ as functions of the electric conductivity $\sigma$.

As previously mentioned, the contributions to the magnetic field come from charged particles in spectators, internal currents induced by Ohm’s law and CME. Thus, it is necessary to explore how each effect affects the evolution of the magnetic field. Figure 3 depicts the temporal evolution of the magnetic field $B_x$ and $B_y$ at the point (0, 0, 0) with the electric conductivities $\sigma =0, 0.5, 5, {20}\; {\text {MeV}}$. Firstly, it is indicated that high energy heavy-ion collisions generate a strong magnetic field, reaching its peak value within a short moment. Secondly, a significant change in the propagation mode of the magnetic field is observed before and after the time of collision. The magnetic field $B_y$ attains its maximum value $B^{max}_y$ in the vacuum within approximately ${0.3} \; {\text {fm}}/c$. After the collision, the magnetic field persists for an extended duration of $3{-}{5} \; {\text {fm}}/c$ in the QGP conductive medium, with reduction of magnetic intensity being suppressed. After that, a wake magnetic field is nearly time-independent in QGP. The time profile needed for the magnetic field to decrease to $0.1B^{max}_y$ for various conductivity levels is displayed in Fig. 4, which can be considered as the lifetime of the effective magnetic field. A comparison of the magnetic field decay rates under different electric conductivities reveals that the time evolution process of the magnetic field is mainly regulated by electic conductivity, meanwhile, the extended lifetime provides sufficient evolution time for the interaction between magnetic field and QGP.

**Figure 5:**
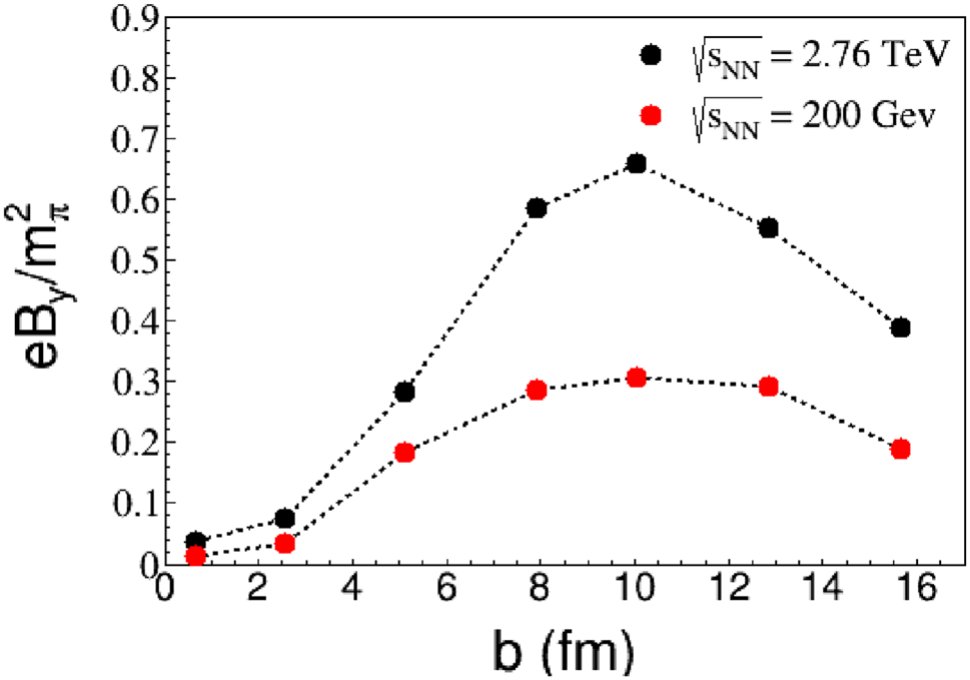
Magnetic filed $B_y$ at $t = {0.3} \; {\text {fm/c}}$ as functions of the impact parameter *b*. The escalation in collision energy enhances the magnetic field.

In high energy heavy-ion collisions, the intensity of the magnetic field can be significantly dominated by impact parameters and the collision energy. In Fig. 5, we make a comparison of the magnetic field generated at $t={0.6} \; {\text {fm}}/c$ for different impact parameters and two collision energies. The results show that the magnetic field initially increases, then decreases with increasing impact parameters. In Au+Au collisions, the maximum magnetic field value occurs at $b={10} \; {\text {fm}}$, which corresponds to the radius of the Au nucleus. As the collision parameter increases from 0 to ${10} \; {\text {fm}}$, the system’s asymmetry increases, leading to an increased magnetic field intensity in the *y* direction. However, when the parameter *b* exceeds the nuclear radius, most of the nucleons are distant from the collision regione, causing a decrease in the magnetic field intensity. The dependence of field intensity on impact parameter suggest that semi-symmetric collisions are the best way to test the effects related to magnetic fields in RHIC and HLC experiments. Additionally, the magnetic field’s dependence on collision energy is evident. As the energy increases, the nucleus’s valence charge moves faster, resulting in a stronger generated magnetic field.

**Figure 6:**
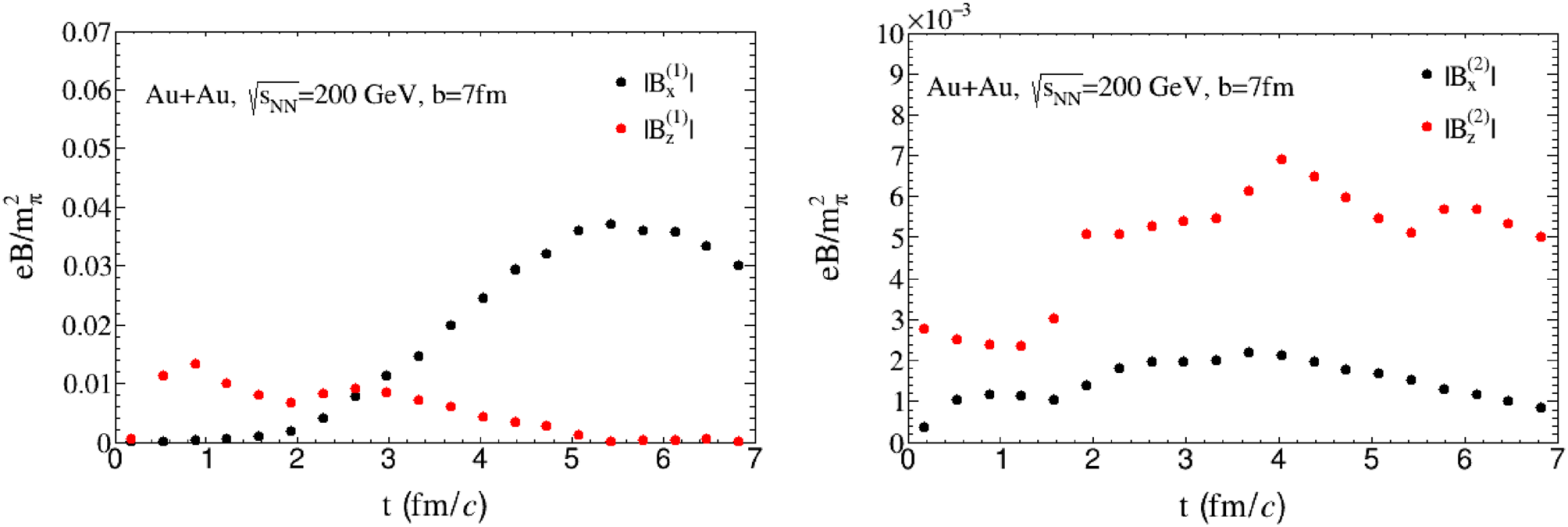
The time evolution of magnetic field $\varvec{B}^{(1)}$ (left panel) and $\varvec{B}^{(2)}$ (right panel) at the point $\varvec{r}=0$, $\sigma = {5.0}\; {\text {MeV}}$, $\sigma _{\chi } = {0.2}\; {\text {MeV}}$ for $\mathrm{Au+Au}$ collision at $\sqrt{s_{NN}}={200}\; {\text{GeV}}$ and $b = {7} \; {\text {fm}}$.

The temporal evolution of the *x*, *z* component of $\varvec{B}^{(1)}$ and $\varvec{B}^{(2)}$ at the original point (0, 0, 0) is depicted by Fig. 6. Here, we set $a_{x}={2.2}\; {\text {fm}}$, $a_{y}={3.0} \; {\text {fm}}$, $c_{s}=0.3$ and $\sigma _{\chi }={0.2}\; {\text {MeV}}$. As previously discussed, in the absence of diffusion effect and chiral conductivity of quark-gluon plasma, the collision structure’s geometric symmetry only generates a strong magnetic field $\varvec{B}^{(0)}$ in the *y* direction and fluctuations in the *x* direction, with no electromagnetic background in the *z* direction of the beam. However, when considering the diffusion effect and chiral conductivity, the situation changes. These two effects lead to the generation of new magnetic fields in the *x* and *z* directions, especially in the *z* direction, where there was previously no electromagnetic field background. Nevertheless, these two effects are relatively weak compared to the magnetic field in the *y* direction. The magnetic field resulting from the diffusion effect of the quark-gluon plasma accounts for around $10\%$ of the total magnetic field, while the chiral conductivity contributes less than $1\%$. Despite its weakness, spectral analysis has recently been proposed as a potential method for detecting CME effects in experiments, which may be feasible for the detection of magnetic fields generated by the chiral conductivity^42^.

## Conclusion

In summary, we utilize the AMPT model to simulate the non-central collision of $\mathrm{Au+Au}$ nucleis at $\sqrt{s_{NN}}={200}\; {\text{GeV}}$ and examine the distribution of the magnetic field generated during the collision. We assume that the plasma diffusion and the chiral magnetic effect on the magnetic field are only perturbations. By utilizing the Green function method, we obtain an analytical expression of the magnetic field, considering the electric conductivity, the chiral conductivity, and the plasma diffusion. Our analysis demonstrates that under the QGP conditions of $\sigma ={5}\; {\text {MeV}}$, the magnetic field’s lifetime can reach up to approximately $0.8{-}{1.2} \; {\text {fm}}/c$, with a maximum strength of about $2{-}{5}{\text {m}_{\pi }^{2}}/e$ in various collision events, and there will be a corresponding increase in the lifetime of the magnetic field as conductivity enhances. The spectator nucleons generate the primary strength of the magnetic field. Subsequently, the magnetic field expands in the nuclear matter formed by the participants on the horizontal plane, and the electric conductivity will prolong the lifetime of magnetic field. The impact of plasma diffusion and the chiral magnetic effect is gradually reflected in the later stages of the collision, including the emergence of new magnetic fields in the *x* and *z* directions. Nonetheless, the overall effects remain weak relative to the maximum magnetic field value devoted by spectators, and their total contributions are less than $10\%$.

## Data Availability

The datasets generated during and/or analyzed during the current study are available from the corresponding author on reasonable request.
